# Equine-assisted learning and leadership transformation: an exploratory qualitative study of workplace behavior

**DOI:** 10.3389/fvets.2025.1700029

**Published:** 2025-11-25

**Authors:** Rubentheran Sivagurunathan, Abdul Rahman bin S. Senathirajah, Linkesvaran Sivagurunathan, Lawrence Arokiasamy, Sayeeduzzafar Qazi, Rasheedul Haque, Yanmei Su

**Affiliations:** 1 Faculty of Business and Communications, INTI International University, Nilai, Malaysia; 2 Faculty of Management, Shinawatra University, Pathum Thani, Thailand; 3 Wekerle Business School, Budapest, Hungary; 4 Department of Management, Faculty of Science, Management and Computing, Universiti Teknologi Petronas, Seri Iskandar, Malaysia; 5 University of Business and Technology, Jeddah, Saudi Arabia; 6 School of Management & Business, MILA University, Nilai, Malaysia; 7 Faculty of Education, Shinawatra University, Pathum Thani, Thailand

**Keywords:** experiential learning, leadership, animal-assisted interventions, equine-assisted learning, organizational psychology, workplace behavior, sustainable growth

## Abstract

**Background:**

This study explores how equine-assisted leadership development (EALD) interventions activate experiential processes that reshape leaders’ self-concept, relational schemas, and behaviors. A conceptual model is proposed to explain how non-verbal interaction with horses catalyzes transformational learning.

**Methods:**

A qualitative exploratory design was employed to examine leaders’ experiences over 12 months following reintegration into their workplaces. Eight leaders (*n* = 8) attended a 5-day EALD program, engaging in “join-up” exercises with horses. Data were analyzed through reflexive thematic analysis to identify leadership learning and transformation patterns.

**Findings:**

The reflexive thematic analysis produced three interrelated themes: (1) embodied self-awareness and leadership reflection, (2) transformative learning and behavioral change, and (3) relational leadership and trust-based engagement—which together describe a developmental pathway from immediate embodied feedback to sustained workplace change.

**Originality/value:**

This is the first Malaysian study to show how structured human–animal interaction can elicit sustained leadership shifts. By positioning horses as non-verbal feedback systems, the research highlights embodied, affective, and relational dimensions of leadership development often overlooked in cognitive or skills-based models.

## Introduction

In today’s turbulent business environment, organizations face mounting internal and external pressures that underscore the critical need for effective leadership (1, 2). Leaders with strong skills, behaviors, and values hold a competitive advantage, as they can guide teams effectively through fast-changing environments to achieve organizational goals (3, 4). As a result, leadership development has become a strategic priority for sustaining competitive advantage, with growing recognition that program design must align with both individual needs and broader organizational contexts (5, 6).

Program effectiveness is typically evaluated by examining measurable, sustainable changes in leader–follower relationships and the resulting improvements in organizational outcomes (7). However, their success ultimately depends on integration into daily practice. Development must be embedded throughout organizational operations and supported consistently from senior executives to frontline managers (8). Traditional corporate leadership development has long been grounded in cognitive and behavioral learning models, typically involving coaching, mentoring, case studies, competency frameworks, and classroom instruction (9, 10). These methods emphasize knowledge transfer, rational decision-making, and observable behaviors addressed through structured teaching and feedback.

Experiential approaches provide a robust alternative by centering on action, reflection, emotional engagement, and embodied practice. Drawing on experiential learning theory (11), growth occurs through lived experience rather than passive information transfer. These individualized, recursive, and systemic approaches require participants to engage with beliefs, anxieties, and relational patterns. Examples include outdoor adventure programs, equine-assisted interventions, martial arts, and somatic coaching. Introducing discomfort, unpredictability, and social feedback mirrors real leadership challenges while fostering tacit knowledge, emotional intelligence, situational awareness, and personal transformation (12)–(13). Experiential programs also encourage shared meaning-making, aligning with complexity theory and adaptive leadership frameworks emphasizing non-linear, context-specific learning (14). Symbolic and metaphorical elements, such as horseback riding representing balance, emotional regulation, and strategic focus, promote deep personal insight and sustained behavioral change (15).

The growing interest in experiential methods aligns with the rise of embodied leadership, which highlights physical, emotional, and somatic dimensions as central to leadership enactment (16). This perspective challenges conventional models that privilege rational thought and verbal communication over bodily intelligence, intuition, and non-verbal dynamics (17, 18).

Embodied leadership draws from neuroscience (19), somatic psychology (20), mindfulness studies (21), and phenomenology (22). These perspectives show how posture, breathing, tone, and movement influence self-regulation, relationships, and ethical decision-making. The body carries memory, cultural conditioning, and lived experience, shaping leadership responses.

Organizations increasingly recognize the need for leaders who can remain resilient under pressure, build authentic relationships, and navigate complexity with integrity (23). Embodied practices have been shown to enhance resilience, empathy, and presence, thereby equipping leaders with these critical capabilities (24). Psychological development research shows that leaders expand their ability to manage complexity and ambiguity as they move through stages from dependent orientations to self-authoring and self-transforming mindsets (25, 26). These transitions, often marked by emotionally charged “disorienting dilemmas,” can catalyze identity reconstruction and transformational learning (27).

Experiential learning emphasizes knowledge gained through direct engagement and reflection. Almeras and Bresciani (28) note that adult learning extends beyond classroom settings, including problem-solving and reflective processes. Kolb’s experiential learning cycle—concrete experience, reflective observation, abstract conceptualization, and active experimentation, provides a strong theoretical foundation for Equine-Assisted Learning (EAL), as interaction with horses fosters episodic memory, self-awareness, and leadership capacity (11).

Equine-assisted programs (EAP) encompass therapeutic, educational, and developmental practices that leverage the horse’s sensitivity to human behavior and emotion. Four modalities are commonly recognized: Equine Facilitated Psychotherapy (EFP), which integrates equine interaction with psychotherapy to promote trust and resilience (29–31); Equine-Assisted Learning (EAL), which uses horse–human interaction to develop personal and leadership skills (32); Therapeutic Horseback Riding (THR), which improves motor coordination, confidence, and well-being through riding (33); and Hippotherapy, a therapy-led intervention that employs equine movement to enhance motor and sensory functions (34). Within this spectrum, EAL is particularly relevant for leadership development.

These dynamics are extended in organizational contexts through Equine-Assisted Leadership Development (EALD). Research shows that EALD interventions combining equestrian exercises, team-building, and cross-cultural learning enhance leadership effectiveness and interpersonal sensitivity (35). By integrating Kolb’s experiential cycle with equine interaction, EAL fosters leaders who are more adaptive, emotionally attuned, and capable of influencing others through presence rather than positional authority (36, 37).

Over time, this iterative process enables leaders to reconstruct their perspectives on control, trust, and relational presence. These shifts are subsequently integrated into workplace practices, fostering enduring behavioral changes, including greater sensitivity to non-verbal communication, reduced reliance on coercive strategies, and stronger trust-building capacities. Hence, this study was guided by the following research questions:

1. How do leaders articulate the ways in which their leadership styles and skills have evolved as a result of participating in an EALD program?
2. Which aspects of learning from the program do leaders identify as most valuable, and how have these been incorporated into their leadership practices?
3. In what ways does participation in the EALD program shape the dynamics between leaders and their followers in the workplace?

## Methods

### Participants

The study population comprised 50 individuals participating in the “Horseback Therapeutic and Leadership Development Program” at Pusat Ekuin Ladam Merah, Malaysia. All participants took part voluntarily. All participants were advised that they could withdraw from the study at any time, refuse to respond to certain questions, or opt out of follow-up.

All leaders attended the 5-day EALD program in 2023, which included practical “join-up” exercises with horses. The participants worked in the financial management sector in Melaka, Malaysia, and each supervised teams ranging from 10 to 20 employees.

Prior to the program, participants were asked about their previous experience with horses during the registration process. Six participants reported no prior equine experience, while two participants indicated limited recreational exposure. None had formal training or extensive experience in horsemanship. This information was noted to account for potential variability in initial comfort levels and engagement during the program.

### Design

Given the exploratory nature of this project, a qualitative methodology was adopted to capture the nuanced perspectives of leaders regarding the value of EAL for leadership development in the financial management sector.

A purposive sampling strategy was employed to select participants from the pool of 50 eligible leaders who had completed the EALD program at least 12 months prior. Eight participants (*n* = 8) were selected to allow for sufficient depth of qualitative inquiry while maintaining feasibility for detailed thematic analysis. This sample size aligns with established qualitative research principles, which emphasize data richness and depth of insight rather than statistical generalizability (38). Interviews were conducted sequentially, with data saturation—defined as the point at which no new themes or codes emerged. This approach is consistent with recommendations by Guest et al. (39), who found that saturation can often be reached within six to twelve interviews in focused qualitative studies. By adopting this strategy, the study ensured both analytic sufficiency and methodological rigor.

### Materials

The EAL program delivered by the Pusat Ekuin Ladam Merah was developed to improve fundamental leadership skills in business leaders. The program fostered skills including confidence, effective communication, resilience, working in teams and team bonding, problem-solving, and trust. Ten specialized EAL modules were carried out with the leaders.

The EAL program was facilitated by a certified team comprising two accredited practitioners and one senior equestrian coach certified by the Equestrian Association of Malaysia (EAM). All facilitators had more than ten years of experience in delivering equine and therapeutic programs.

Before each session, participants received a structured safety briefing that included appropriate handling techniques, reading equine body language, emergency procedures, and guidelines for respectful and ethical engagement with horses. Both facilitators and stable staff supervised all activities to ensure participant safety throughout the program.

Equine welfare was prioritized at every stage. Horses were selected for their calm temperament, and interaction periods were kept short to prevent fatigue. They were provided regular veterinary checks, adequate nutrition, and rest periods between sessions. All procedures adhered to internationally recognized animal welfare standards and ethical guidelines for equine-assisted interventions.

### Procedures

The research team interviewed individuals via face-to-face and online video, using a semi-structured set of guiding questions, as referred to in Supplementary Appendix A. All interviews were conducted by the lead researcher, who had prior qualitative research training and experience in EAL contexts. The same interviewer conducted all sessions to ensure consistency in data collection. Each interview lasted between 40–60 min and was designed to obtain rich information from participants. All interviews were recorded after obtaining consent from the participants to confirm correct transcriptions and thereby ensure reliability. All information was safely kept and handled in strict compliance with institutional ethical approval and international data protection regulations. Given the small sample size, no detailed demographic information about educators’ faculties or professional roles was disclosed in the reporting of results to reduce the risk of identifying participants.

All research data were anonymized immediately after transcription of the interviews. Therefore, participants were told that any direct quotes might be used in future publications, but would not be tied to identifiable information. Personal data were only stored for three months before it was securely deleted. Anonymized interview and consent forms were securely stored in password-protected files. This research was approved by the Inti International University Research Ethics Committee, and all participants were treated in accordance with institutional guidelines and international standards for research on human subjects.

### Analytic strategy

The interview transcripts were analyzed using thematic analysis as outlined by Braun and Clarke (38). Thematic analysis is a flexible yet rigorous framework for identifying, analyzing, and reporting patterns of meaning across qualitative datasets. For this study, the reflexive TA approach was employed, as it emphasizes the active role of the researcher in interpreting the data and constructing meaningful insights.

After transcription, the lead researcher conducted an initial familiarization phase by repeatedly reading the transcripts and noting early impressions. In the second phase, initial codes were generated inductively, line by line, to capture meaningful segments of data related to participants’ experiences of the EALD program. Two members of the research team developed a preliminary coding framework collaboratively.

In the third phase, codes were reviewed and refined iteratively. Similar codes were grouped, collapsed, or expanded into subcodes to capture nuances across participants’ narratives. Coding was supported by manual annotation and audit trails to ensure transparency in analytic decision-making.

Two researchers independently coded the same subset of transcripts to enhance the analytic rigor. Coding differences were discussed until consensus was reached, and the refined coding framework was then applied to the remaining data. Peer debriefing meetings were held at two key points to challenge assumptions, examine code boundaries, and strengthen thematic coherence.

The research team engaged in ongoing reflexive practice throughout the analysis to mitigate researcher bias. As several researchers are affiliated with the institution delivering the program, analytic procedures were intentionally structured to reduce bias, including involvement of an independent team member not directly engaged in program delivery. The lead researcher maintained a reflexive memo documenting positionality, assumptions, and analytic decisions. This process ensured that the interpretation remained grounded in the data rather than shaped by prior expectations.

Finally, themes were defined, named, and supported with representative exemplar quotes to demonstrate analytic depth and transparency. A coding framework and sample quotations are provided in Supplementary Appendix B to illustrate how raw data segments were transformed into codes, subthemes, and overarching themes.

## Results

The thematic analysis yielded three interrelated themes that capture how participation in the EALD program shaped participants’ leadership perspectives and practices. These themes are:

- Embodied self-awareness and leadership reflection
- Transformative learning and behavioral change
- Relational leadership and trust-based engagement

These themes map directly to the study’s three research questions, addressing how participants described their leadership evolution, identified valuable aspects of the program, and integrated these into leader–follower dynamics.

### Results of research question 1

#### Theme 1: Embodied self-awareness and leadership reflection

The analysis revealed that one of the most significant outcomes of the EALD program was a heightened sense of self-awareness among participants, particularly concerning their leadership presence, communication style, and emotional regulation. Interactions with horses served as a mirror for participants’ internal states, providing real-time, non-verbal feedback that made them conscious of how their behavior and energy influenced their environment.

Participants described becoming more intentional in how they projected themselves as leaders. Many recounted moments when the horse’s lack of response prompted them to adjust their posture, tone, or emotional state to re-establish connection. This reflective process encouraged a deeper awareness of their own leadership tendencies and their impact on others.

For several participants, this embodied experience represented the first time they had physically experienced the consequences of their leadership style without verbal feedback. This immediacy triggered self-reflection in a way that traditional classroom learning could not. Participants linked this new awareness directly to their leadership behaviors in the workplace, noting that they became more attuned to their emotional states, communication patterns, and non-verbal cues when interacting with their teams.

These findings illustrate how EAL fosters embodied leadership reflection, aligning with Kolb’s experiential learning theory in which concrete experience leads to reflective observation and subsequent behavioral change. Participants articulated a shift from unexamined habitual behaviors toward more deliberate, self-aware leadership practices through this process.

A summary of key subthemes and illustrative participant expressions for this theme is presented in Table 1.

**Table 1:** Subthemes and exemplar quotes for Theme 1.

Subtheme	Illustrative codes/participant expressions	Representative quotes
Recognizing embodied presence	Safe space; reading body language; sensing energy; calming oneself	“The horse reacted to my energy before I even said anything.” (P4)
Leadership style reflection	Realizing aggressiveness; adjusting tone; intentional posture	“I realized that I was being too dynamic with my team. The horse did not respond until I softened up.” (P2)
Emotional self-awareness	Awareness of tension; anxiety management; calming influence	“It made me aware of my emotions… the horse mirrors everything.” (P1)
Reflective insight	Leadership identity; understanding personal triggers; growth orientation	“It made me think about how I come across as a leader.” (P6)

### Results of research question 2

#### Theme 2: Transformative learning and behavioral change

Participants identified a range of practical and personal changes that occurred after completing the EALD program, reflecting a transformation of leadership behavior rather than a temporary learning effect. Participants developed new awareness of their communication habits, leadership tone, and emotional regulation through their engagement with horses. Over time, these insights translated into intentional, sustained behavioral changes in their workplace practices.

A dominant subtheme was the shift toward calmer and more adaptive communication styles. Several participants described how the immediate feedback from the horses, often responding better to calm, intentional leadership than forceful behavior, prompted them to alter how they engaged with their teams.

Participants reported that these changes became part of their daily leadership routines, leading to improved workplace interactions and a stronger sense of personal leadership presence. Many emphasized that the program’s impact was not short-lived but continued months after completion.

Another key subtheme was the spillover of behavioral change beyond the workplace. Participants explained that their self-regulation skills, particularly patience, calmness, and emotional awareness, also influenced their personal and family relationships.

These findings reflect a transformative learning process in which participants moved from reflective awareness (Theme 1) to enacting new behaviors that enhanced both their leadership practice and broader interpersonal relationships. This trajectory aligns with Kolb’s experiential learning model, where reflection leads to active experimentation and concrete behavioral change.

A summary of subthemes and illustrative participant expressions for this theme is presented in Table 2.

**Table 2:** Subthemes and exemplar quotes for Theme 2.

Subtheme	Illustrative codes/participant expressions	Representative quotes
Behavioral shifts in communication	Speaking calmly; active listening; being patient	“After the program, I speak less aggressively and listen more carefully to my team members.” (P7)
Adaptive leadership	Tailoring approaches; reading situations; adjusting strategies	“I learned that I cannot use the same approach with everyone on my team.” (P4)
Sustained personal transformation	Lasting changes in work behavior; improved confidence	“Even after a year, I still practice what I learned from the horse.” (P3)
Personal life spillover	Applying lessons at home; emotional regulation outside of work	“It’s not only work. Now I’m more patient with my family now too.” (P8)

### Results of research question 3

#### Theme 3: Relational leadership and trust-based engagement

The third major theme to emerge from the data highlights how participation in the EALD program led to shifts in leader–follower dynamics, characterized by increased trust, openness, and collaborative engagement. Participants described how the process of gaining a horse’s trust during the program prompted them to reflect on their own leadership approaches and the ways they build trust with their teams.

Many noted that learning to lead a horse through calm, clear, and consistent communication made them reconsider the importance of psychological safety and relational presence in their leadership practice.

Participants emphasized that these shifts manifested in more open communication, improved team relationships, and greater willingness among team members to engage and contribute. Leaders described their teams as “more open, less defensive, and more cooperative” after they intentionally changed their own behavior.

An important subtheme in this area was modeling leadership behavior. Participants recognized that how they showed up as leaders set the tone for their teams. Calmness, empathy, and consistency increased mutual respect and stronger team cohesion.

These findings suggest that EALD experiences foster relational leadership capabilities, in which trust and authentic engagement become central to leader–follower interactions. This theme reflects the relational dimension of experiential learning—how internal transformation (Themes 1 and 2) manifests externally through relationships with others.

A summary of subthemes and illustrative participant expressions for this theme is presented in Table 3.

**Table 3:** Subthemes and exemplar quotes for Theme 3.

Subtheme	Illustrative codes/participant expressions	Representative quotes
Trust-building	Mutual respect; reading signals; building rapport	“Once I gained the horse’s trust, I realized I need to earn the same trust from my followers at work.” (P5)
Relational engagement	Empathy; openness; psychological safety	“Now my team opens up to me more as I think it’s because I lead with more patience.” (P3)
Team collaboration	Supportive environment; reduced conflict; shared purpose	“I’ve noticed that the team dynamic is more cooperative since I changed how I lead.” (P2)
Modeling leadership behavior	Leading by example; consistency; mutual accountability	“I try to show the same calm presence with my team that I learned from handling the horse.” (P7)

## Discussion

This study examined how participation in an EALD program influenced leaders’ self-concept, behaviors, and leader–follower dynamics twelve months after program completion. The reflexive thematic analysis produced three interrelated themes: (1) embodied self-awareness and leadership reflection, (2) transformative learning and behavioral change, and (3) relational leadership and trust-based engagement. Together, they describe a developmental pathway from immediate embodied feedback to sustained workplace change. Below, we discuss each theme in relation to theoretical perspectives and prior findings, consider practical and theoretical implications, and outline limitations and directions for future research.

### Theme 1: Embodied self-awareness and leadership reflection

The first theme highlights how non-verbal, cross-species interaction served as an embodied mirror, triggering heightened self-awareness of leaders’ presence, tone, and non-verbal signals. Participants described immediate affective dissonance when horses failed to respond to habitual, forceful behavior and reported adjusting posture, breathing, and affect to re-establish connection. This process aligns with embodied leadership literature that emphasizes the role of somatic cues in leadership enactment (16, 40) and supports Kolb’s assertion that concrete experience catalyzes reflective observation.

This finding underscores the central role of engagement in participants’ reflections, with join-up perceived as a sustained and meaningful encounter. Within the broader literature, join-up is described as a technique that fosters relational connection between horse and participant, representing the most fundamental form of leader–follower interaction (41, 42). Moreover, in a study conducted by Stock and Kolb (43), they emphasized that acknowledging and managing fear during equine-assisted activities enhances confidence and resilience. Similarly, Gehrke et al. (35) observed that equine interactions often evoke initial fear responses, which, once addressed, promote growth in self-awareness. These findings suggest that the majority of the participants experienced join-up not only as a practical exercise but as a profound and memorable form of engagement, echoing Miller’s (44) assertion that positive equine interaction can foster meaningful leadership insights.

These findings corroborate a prior EALD study showing that horses provide salient, non-verbal feedback that surfaces tacit aspects of human behavior (43). The immediacy of equine responses appears to make implicit leadership habits visible, prompting reflective questioning that participants reported rarely encountered in traditional classroom or coaching classes. Williams (45) identified interaction with horses as a fundamental element of EALD, while Jung et al. (46) argued that establishing a bond with the horse is a necessary precursor to any therapeutic or developmental process. For participants, the ability to effectively engage with horses translated into enhanced capacity to build relationships with followers in workplace settings. In short, the horse functions as an embodied feedback system that accelerates self-observation and highlights the somatic dimensions of leadership presence.

### Theme 2: Transformative learning and behavioral change

The second major theme reflects how the embodied insights gained during the EAP translated into tangible and sustained behavioral change in participants’ leadership practice. After becoming more aware of their internal states, participants described actively adjusting their communication styles, emotional responses, and relational approaches in the workplace. The importance of behavioral engagement is well-documented in the literature as a critical competency for effective leadership. Gemeda and Lee (23) highlighted engagement as a central factor in enhancing leadership effectiveness, while Hernandez et al. (47) emphasized its role in shaping leader–follower dynamics. Stock and Kolb (43) similarly observed that participants applied enhanced communication skills gained from EAL in the workplace, producing favorable outcomes. Effective communication, therefore, serves as the foundation for fostering behavioral engagement. Elif (32) further reinforced that cultivating team relationships is essential within organizational contexts, as engagement is central to building strong leader–follower bonds.

After becoming more aware of their internal states, participants described actively adjusting their communication styles, emotional responses, and relational approaches in the workplace. Participants consistently reported becoming calmer, more patient, and more adaptive in their leadership style after completing the program. Schultz et al. (48) observed that horses were more responsive to participants who displayed lower levels of aggression, reinforcing the principle that relational attunement is fostered through calm and respectful engagement. Similarly, Braun et al. (36) found that participants exhibited measurable decreases in aggression following involvement in equine-assisted activities, supporting the notion that horses can facilitate behavioral regulation. This shift is consistent with the core principles of transformative learning theory, where disorienting experiences prompt reflection, perspective transformation, and behavioral change (49).

Participants reported sustained adjustments in their everyday interactions. Many indicated that they no longer relied on forceful or directive approaches to influence others but instead adopted calmer, trust-based communication. Leaders described an increased ability to regulate their emotions, communicate with clarity, and build trust in their teams over time. Burgon (50) described this ability as a skill developed during the join-up exercises, where participants learn to manage unexpected events. Sullivan and Hemingway (51) similarly noted the calming influence of horses on individuals, contributing to a more positive demeanor during challenging interactions. More recently, Ayala et al. (52) confirmed the therapeutic role of horses in reducing anxiety and promoting calmness, thereby improving communication. Within leadership contexts, the ability to remain composed in uncertain circumstances enhances leaders’ capacity to guide teams effectively.

This behavioral change extended well beyond the immediate post-training period, with several participants reporting that the lessons learned through their equine experiences were still influencing their leadership behavior a year later. This resonates with Gehrke (53), who reported that students could articulate deep personal reflections even after 24 months, attributing these recollections to positive developmental outcomes. Stock and Kolb (43) further support this observation, noting that participants retained sequential learning experiences over extended periods, reinforcing the enduring nature of experiential learning.

The join-up activity again emerged as a critical learning moment that bridged awareness and behavioral experimentation. Through iterative attempts to gain the horse’s trust, participants learned to regulate their energy, slow down their responses, and adopt a leadership style grounded in presence rather than control. Participants noted that the shift from aggressive to calm and intentional communication with the horse carried over into their workplace interactions, where they observed improved team responses and less resistance. This observation resonates with Meola (54), who reported that EALD participants demonstrated greater empathy, more transparent communication, a more optimistic attitude, and a calmer demeanor in workplace interactions. Similarly, Gehrke (53) documented cases where previously aggressive participants adjusted their behavior after recognizing alternative approaches, leading to improved teamwork and relational dynamics.

These findings can be understood through Kolb’s experiential learning cycle, where participants engaged in concrete experience (interacting with the horse), reflected on the outcomes, conceptualized new approaches, and then actively applied them in real-world contexts. This iterative learning process supports existing scholarship on the efficacy of experiential modalities in leadership development (55).

The embodied nature of the program made the learning more memorable, impactful, and applicable over time, unlike short-term cognitive leadership workshops that often fail to translate into practice. This reinforces arguments in the literature that embodied experiences create deep cognitive-emotional integration, leading to more durable shifts in behavior (56, 57).

Taken together, these findings provide strong support for the idea that EALD is not merely an awareness-building intervention but can foster long-lasting behavioral transformation. By linking emotional regulation, authentic communication, and adaptive leadership practice, EAL helps leaders internalize new ways of leading that extend well beyond the training environment.

### Theme 3: Relational leadership and trust-based engagement

The third major theme illustrates how individual transformation experienced through the EALD program manifested in leader–follower relationships. After increasing their self-awareness and transforming their behavioral approaches, participants described stronger, more trust-based, and collaborative interactions with their teams. Leaders reported greater self-awareness of their influence on others, leading to a reduction in authoritarian and reactive behaviors, and the development of more open, trusting, and empathetic relationships with their team members. This observation aligns with findings by Rajfura and Karaszewski (58), who reported that leaders participating in EAP demonstrated greater cooperativeness and provided moral support to their subordinates upon returning to the workplace. This suggests that leaders not only adapted their behavioral style but also redefined their role by offering support less authoritatively and more collaboratively.

Many participants drew direct parallels between the trust-building required with the horse and the trust-building required with followers in their professional settings. Horses require calm, congruent, and authentic communication to willingly engage with a leader. Similarly, participants recognized that trust from their followers must be earned rather than demanded. Several participants described realizing that trust cannot be commanded as it has to be built through consistent, calm, and authentic presence. This aligns closely with relational leadership theory, which conceptualizes leadership as a social process rooted in trust, respect, and mutual engagement (59).

Participants reported that these relational shifts resulted in observable changes in team dynamics, including more open communication, greater follower initiative, and reduced defensiveness. Leaders observed that as they changed their own behavior, their followers became more receptive, communicative, and collaborative. This reflects social learning processes in which followers model and mirror leader behaviors, contributing to a more psychologically safe and participatory environment. These results support prior research showing that EAP can foster trust-based leadership behaviors (53, 60). The join-up activity again played a key role. Just as horses respond to calm leadership, emotional congruence, and presence, participants found that applying these same qualities in the workplace improved team engagement.

This trust-building mechanism resonates strongly with relational and authentic leadership frameworks, which emphasize the importance of emotional connection, trustworthiness, and transparent communication (59, 61). The embodied learning context of EALD appears to strengthen these relational skills not through didactic teaching but through direct experience of building trust with a non-verbal partner, which then transfers to human interactions. Leaders observed that as they became more attuned to their own emotions and behaviors, followers responded in kind, creating a cycle of mutual trust and respect. The finding further supports Pohl’s (62) assertion that leaders are increasingly expected to cultivate empathetic, individualized connections with their followers. This suggests that EALD may not only enhance individual leader capability but also activate relational processes within teams. Such ripple effects are rarely documented in conventional leadership training programs.

Taken together, these findings reinforce the notion that trust is the central aspect of leadership and that embodied experiential interventions offer a powerful way to develop it. By modeling trust-based engagement through equine interaction, leaders were able to translate those relational skills into their professional settings, fostering stronger and more authentic leader–follower connections.

### Integrating the themes and theoretical contributions

The three overarching themes identified in this study are embodied self-awareness and leadership reflection, transformative learning and behavioral change, and relational leadership and trust-based engagement—together reveal a coherent developmental trajectory through which EALD supports leadership growth. Rather than functioning as isolated insights, these themes represent interconnected phases of an experiential learning pathway toward embodied awareness, behavioral transformation, and relational integration.

While the three themes identified in this study illustrate a clear developmental trajectory in leadership learning, it is important to note that not all participants experienced the EALD program similarly. A few expressed discomfort or difficulty engaging with the non-verbal and embodied nature of the exercises. These divergent experiences underscore that the process may not resonate equally for all leaders and highlight the importance of individual readiness and learning preferences in experiential leadership development.

The study integrates Kolb’s experiential learning cycle by emphasizing the embodied, affective, and somatic dimensions of learning. The horse’s immediate and intuitive response provides a powerful form of concrete experience that triggers reflection and behavioral adjustment. While Kolb’s experiential learning cycle emphasizes the iterative process of concrete experience, reflection, conceptualization, and action, the EALD study advances this framework by incorporating real-time, embodied feedback through equine interaction. This immediate, non-verbal response accelerates reflective processing and behavioral experimentation, offering a more visceral and emotionally anchored learning experience than traditional classroom-based approaches.

One of the central theoretical contributions of this study lies in its articulation of interspecies mirroring as a mechanism of leadership development. Horses respond instinctively to congruence between internal emotional states and external behavioral cues, offering immediate and non-judgmental feedback. Unlike traditional human feedback, which can be deferred, filtered through social norms, or influenced by power dynamics, equine feedback is immediate, non-judgmental, and emotionally attuned. This unique embodied mirroring bypasses cognitive defensiveness and engages participants at a deeper emotional level, thereby accelerating the learning cycle and enhancing self-awareness.

Hence, this study provides a conceptual model known as the EALD Experiential Learning Model to describe how equine interaction creates an embodied feedback loop that fosters leadership development. In this model, leaders engage with horses through authentic, non-verbal communication, encounter immediate somatic feedback, and undergo a process of critical reflection and behavioral recalibration, which in turn translates into human relational contexts. Through join-up exercises, the horse provides embodied feedback by mirroring the leader’s actions and emotional state, thereby revealing subconscious behaviors and attitudes. This embodied learning experience acts as a catalyst for shifts in leadership values and behavioral patterns.

This dynamic contributes to existing theoretical frameworks by bridging embodied, transformative, and relational leadership theories. As recent studies show, transformative learning is often triggered not only by life crises but also by disorienting dilemmas in professional settings that compel learners to reflect on previously held assumptions critically (63, 64). In EALD, the inability to control or coerce the horse provides such a disorienting moment, catalyzing reflection on one’s leadership approach and personal presence. At the same time, the embodied nature of the experience aligns with (17, 65) perspectives on embodied leadership, where leadership effectiveness is shaped not only by cognition but also by bodily awareness and emotional attunement.

Beyond integrating these theories, the EALD model also extends leadership development frameworks. By embedding embodied learning into leadership development, EALD complements and extends existing approaches such as transformational, adaptive, and identity-based leadership. The emphasis on trust-building, authentic presence, and emotional regulation aligns with transformational leadership’s focus on relational influence (66) and adaptive leadership’s emphasis on flexibility and reflection (67). Furthermore, by deepening leaders’ awareness of how they show up in interactions, EALD supports identity work central to authentic and relational leadership development (59, 68).

Taken together, these contributions position the EALD model as a theoretically grounded and distinctive approach to leadership development. It builds upon well-established frameworks like Kolb’s experiential learning cycle and transformative learning theory, while introducing interspecies feedback as a novel mechanism that enhances immediacy, authenticity, and emotional depth. This model also reinforces the growing body of literature emphasizing embodied and relational leadership development as critical complements to traditional cognitive approaches.

It is important to note that these findings are based on participants’ self-reported perceptions rather than direct behavioral observations. While several participants described observable changes in their teams, these accounts were not independently verified. The interpretation of impact should therefore be understood as perceived or experienced change, rather than objectively measured behavioral outcomes.

### Formulation of the research conceptual framework

**Figure 1:**
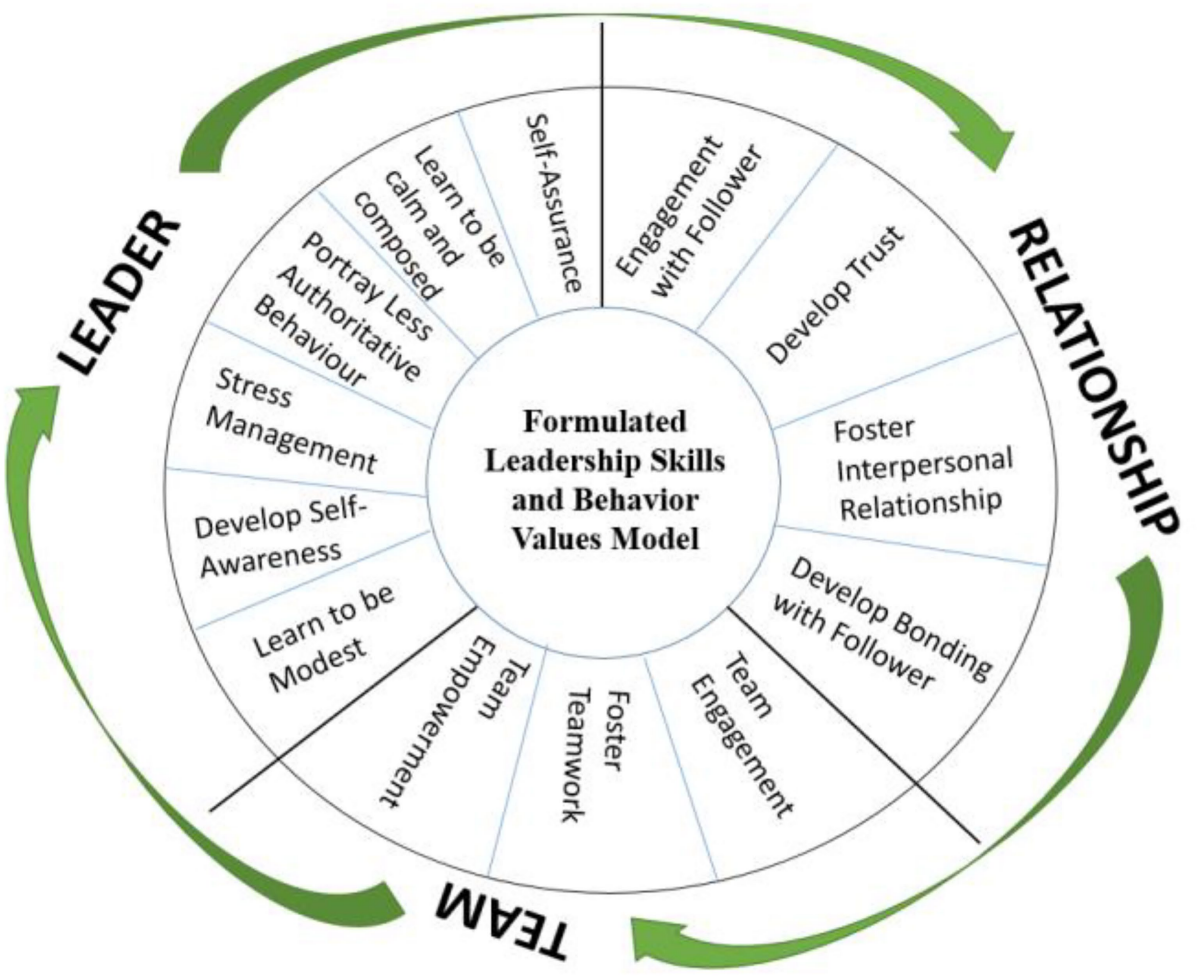
Formulated leadership skills and behavior values framework. Adapted from: Whetten and Cameron (69).

To gain a deeper understanding of the influence of the EALD course on leaders, the leadership skills and behavioral values identified through the three research questions were synthesized into a “Formulated Leadership Skills and Behavior Values Framework” as illustrated in Figure 1. This framework is adapted from Whetten and Cameron’s “Model of Essential Management Skills” and is tailored to reflect the distinctive outcomes of EALD.

The findings across the three research questions indicate that the EALD program fostered a progression of leadership competencies and behavioral shifts. When participants interacted with the horses, they became more aware of the importance of engagement, self-awareness, self-assurance, and modesty. These personal insights extended into their professional roles, where leaders reported enhanced composure and calmness in managing stress and unexpected challenges.

Beyond individual growth, the program significantly influenced workplace dynamics. Leaders reported less reliance on authoritarian approaches, instead adopting more individualized and empathetic interactions with followers. Improvements in communication, trust, and relationship-building supported this transition. Furthermore, the emphasis on collaboration led to strengthened interpersonal relationships and more cohesive teamwork environments.

Collectively, the conceptual framework highlights a dual impact of the EALD course: (i) intrapersonal development through increased emotional regulation and self-awareness, and (ii) interpersonal enhancement through improved trust, communication, and collaborative practices. Together, these outcomes illustrate how experiential learning with horses can be translated into sustainable leadership practices that balance authority with empathy and foster a culture of engagement and shared responsibility within teams.

### Implications of the EALD program on leaders

**Figure 2:**
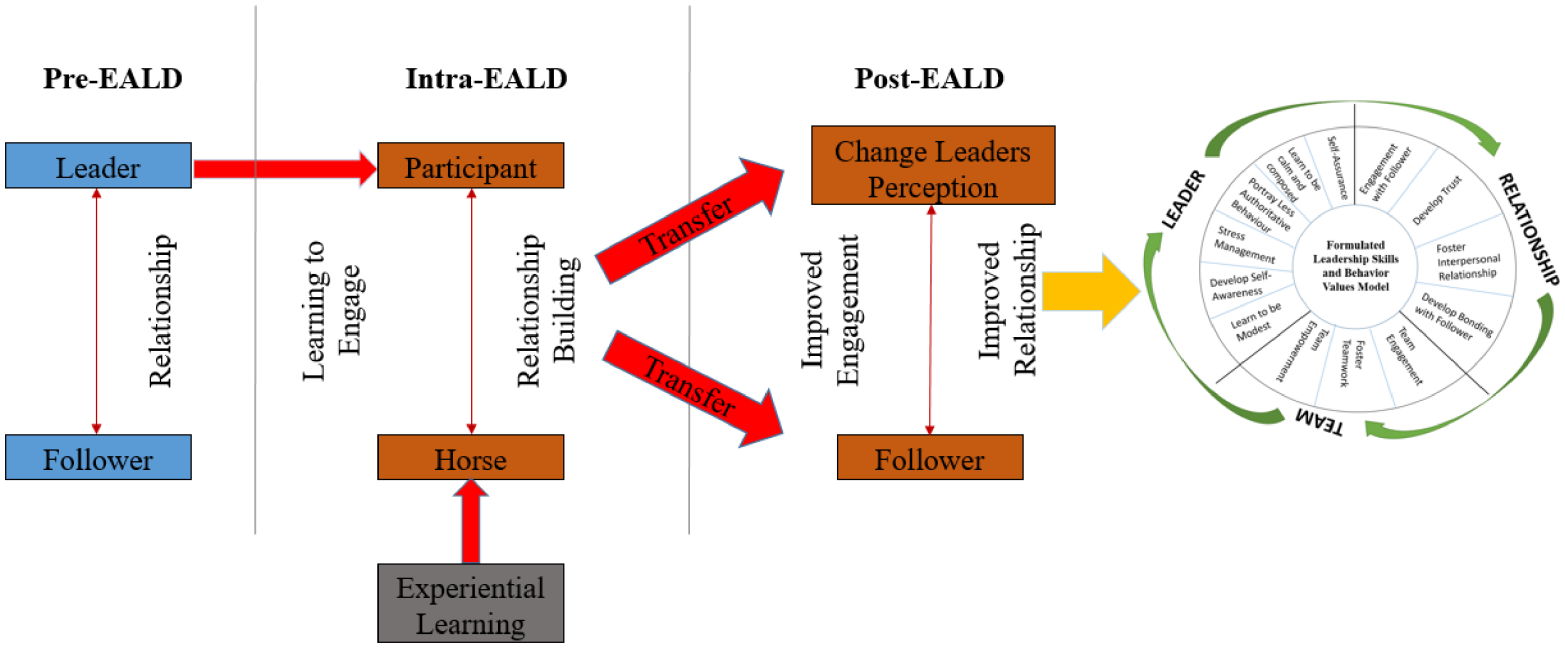
EALD experiential learning model.

The findings of this study revealed a conceptual model—the EALD Experiential Learning Model, which collectively illustrates the developmental trajectory of leaders from their initial state prior to program participation through to the behavioral and relational outcomes observed post-intervention, as shown in Figure 2. This model demonstrates how the EALD program influences leaders’ skills, behaviors, and values, with subsequent effects on their interpersonal interactions and team relationships. The process is underpinned by Kolb’s experiential learning theory, which highlights reflection as a critical mechanism for assisting leaders in navigating complex organizational environments and strengthening workplace relationships.

In the pre-stage of the EALD Experiential Learning Model, the leader is situated within an existing leader–follower system. At this stage, the leader’s skills, behavioral tendencies, and values shape the nature of the leader–follower relationship, which in turn affects both individual and team performance. This stage establishes the baseline from which developmental changes can be observed.

The second phase of the EALD Experiential Learning Model begins with the leader’s participation in the EALD course, where each participant is paired with a horse. Through join-up exercises and other structured interactions, the horse provides embodied feedback by mirroring the leader’s actions and emotional state. As participants engage in this reflective process, they begin to develop greater awareness of how their behaviors influence relational dynamics. The horse’s gradual relaxation and submission serve as tangible indicators of trust and connection, reinforcing the principles of engagement, composure, and adaptability. This embodied learning experience functions as a catalyst for shifts in leadership values and behavioral patterns.

The third phase occurs once participants reintegrate into the workplace. Here, the leader demonstrates tangible behavioral change as a direct outcome of the experiential learning process. Leaders reported greater self-awareness of their influence on others, leading to a reduction in authoritarian and reactive behaviors. Instead, they adopted more open and individualized approaches, fostering stronger interpersonal connections with followers. This shift not only enhanced relational dynamics but also enabled leaders to build trust and empower their teams by encouraging autonomy and participation. Consequently, the EALD program facilitated a more collaborative and trust-based leadership style, with clear benefits for both leaders and their teams.

### Research, practical, and social implications

This research aims to provide participants with opportunities to engage in and reflect on exercises with horses, helping them translate their newly gained abstract knowledge into their professional environments. The goal is to enhance the leader–follower relationships and improve leadership skills, resulting in self-motivation, business growth, organizational change, and positive social and economic outcomes (70).

#### Contribution to leadership development theory

By conceptualizing EAP as a kind of embodied, experiential learning that can foster leadership development, this study fills an important gap in the scholarly literature. Although current studies in EAP provide early ideas on how horse–human interactions affect psychological aspects, very few studies have looked at EAL as a planned, organized intervention for developing leadership potential. This study develops theories of embodied leadership and transformational learning by means of EAL as both a physical discipline and a symbolically rich practice. It adds depth to the discussion by highlighting the integration of somatic intelligence in leadership development.

#### Innovation in experiential leadership practice

The research suggests EAL as an experiential method that could solve major shortcomings in traditional corporate leadership courses. Unlike usual offsite or classroom-based projects, EAL requires presence, trust, composure, and embodied alignment under dynamic, high-focus conditions that reflect the intricate relational and performance expectations put on leaders in modern organizational life. The results of this study could inspire new experiential curricula or executive coaching techniques using EAL or other related embodied modalities to promote leadership development in holistic and sustainable ways.

#### Practical relevance to organizational leadership

Practically speaking, the research offers executive coaches, human resources (HR) experts, and business leaders a new and integrative approach to leadership development. It emphasizes how organized interaction with non-traditional, embodied disciplines can help resilience, focus, relational intelligence, and self-mastery qualities become more and more important in guiding teams, handling crises, and maintaining ethical decision-making in high-stress situations. The results could motivate new routes for leadership training programs, especially those hoping to stand out by means of creative, human-centered, and meaning-rich learning interventions. By presenting a philosophically grounded, empirically rich, and practically relevant framework for leadership transformation via EAL, this study offers a timely and original contribution to leadership development. It questions accepted ideas, provides practical ideas for people creating leadership interventions in the tough corporate environment of today, and opens up fresh avenues for study.

### Limitations of this research

This study has several limitations that should be acknowledged. First, the small sample size and use of purposive sampling limit the transferability of findings to broader leadership populations. The study was conducted within a single sector (the financial management sector in Malaysia) and reflects context-specific experiences that may not fully capture perspectives from other sectors or cultural settings.

Second, the study relied on self-reported accounts collected through retrospective interviews conducted 12 months after the program. While this approach provided valuable long-term insights, it may also be subject to recall bias and social desirability effects, potentially influencing how participants described their leadership behaviors and personal growth.

Another consideration is the potential influence of prior equine experience among participants. Although the majority of participants reported no previous exposure to horses, two individuals had limited recreational experience. These differences may have influenced initial levels of comfort and confidence when interacting with the horses, potentially shaping their experiential learning processes.

Finally, this study acknowledges the potential influence of external factors such as broader organizational dynamics, evolving team structures, or natural leadership maturation over time that may have contributed to the perceived improvements in leadership practice. While participants attributed their growth to the EALD program, it is not possible to fully isolate the program’s effects from these broader influences. Recognizing these factors adds reflexivity and strengthens the critical interpretation of the findings.

### Recommendations for future research

Based on the findings of this study, several directions for future research are recommended to strengthen and expand the existing literature. First, examining the cross-cultural applicability of EAL would help determine whether interspecies feedback mechanisms operate consistently across diverse cultural contexts.

Second, future research could address these limitations by incorporating longitudinal or mixed-method designs, integrating third-party observations, or examining organizational variables to better differentiate the program’s specific impact.

Finally, comparative research should investigate how EAL measures against other embodied modalities, such as improvisational theatre or martial arts, to evaluate the unique contributions and relative impact of non-human feedback in leadership development.

## Conclusion

This study shows that EAL advances leadership development through experiential and embodied processes. Non-verbal interaction with horses offered authentic feedback that generated affective dissonance, encouraging leaders to reconstruct their identity and relational patterns. Interviews indicated lasting improvements in communication, self-awareness, and workplace relationships. The research extends experiential learning and embodied leadership theories, positioning interspecies mirroring as a distinct mechanism for transformational learning. Theoretically, this highlights the value of integrating cognitive, emotional, and somatic dimensions in leadership development. Practically, it demonstrates the potential of EALD programs to cultivate more adaptive, relationally attuned leadership.

## Data Availability

The raw data supporting the conclusions of this article will be made available by the authors, without undue reservation.
