# Supplementary material for: Equine-assisted learning and leadership transformation: an exploratory qualitative study of workplace behavior
Source: Front Vet Sci. 2025 Nov 25;12:1700029. doi: 10.3389/fvets.2025.1700029 (PMC12685683; doi:10.3389/fvets.2025.1700029)
Supplement: Supplementary file 1 [file Table_1.docx]

**Appendix A**

Research Questionnaire

| **Leadership Experience** |
| --- |
| - What is your present role and how long have you held this position? - What is the number of subordinates under your direct/indirect supervision? - How long have you been in a leadership role? |
| **Personal Experience from the Program (RQ 1)** |
| 1. One year has passed. Which aspects of the course continue to be remarkable to you? 2. What insights did you gain about yourself as a result of this particular experience? |
| **Helpfulness and Influence from the Program (RQ 2)** |
| 1. What lessons do you recall that proved beneficial? Conversely, what lessons was deemed unhelpful? 2. What has been effective for you lately? 3. Could you provide an example? How did it benefit you? 4. In what ways have your behaviors or practices been altered as a result of engaging with the program? 5. What was the influence of this experience on you? |
| **Leader and Follower Relationship (RQ 3)** |
| 1. Specifically, what has proven beneficial in terms of your interactions with your followers? |
| 1. Specifically, can you identify any unique practices or approaches that you employ in your interactions with your followers? |
