# Supplementary material for: Equine-assisted learning and leadership transformation: an exploratory qualitative study of workplace behavior
Source: Front Vet Sci. 2025 Nov 25;12:1700029. doi: 10.3389/fvets.2025.1700029 (PMC12685683; doi:10.3389/fvets.2025.1700029)
Supplement: Supplementary file 2 [file Table_2.docx]

**Appendix B**

**Coding Framework and Sample Quotations**

| Raw Data Excerpt (Participant Quote) | Initial Code | Subtheme | Final Theme |
| --- | --- | --- | --- |
| “The horse reacted to my energy before I even said anything.” (P4) | Awareness of body language | Recognizing embodied presence | Embodied self-awareness and leadership reflection |
| “I realized that I was being too forceful with my team. The horse didn’t respond until I softened up.” (P2) | Realizing aggressiveness | Leadership style reflection | Embodied self-awareness and leadership reflection |
| “It made me aware of my emotions… the horse mirrors everything.” (P1) | Emotional awareness | Emotional self-awareness | Embodied self-awareness and leadership reflection |
| “I could feel when I was tense, and the horse would stop moving too.” (P5) | Physical tension awareness | Embodied feedback and self-regulation | Embodied self-awareness and leadership reflection |
| “It made me think about how I come across as a leader.” (P6) | Self-perception and reflection | Reflective insight | Embodied self-awareness and leadership reflection |
| “After the program, I speak less aggressively and listen more carefully to my team members.” (P7) | Behavioral shift in communication | Calmer, intentional communication | Transformative learning and behavioral change |
| “I learned that I can’t use the same approach with everyone on my team.” (P4) | Flexible approach | Adaptive leadership | Transformative learning and behavioral change |
| “Even after a year, I still practice what I learned from the horse.” (P3) | Sustained change | Long-term behavioral transformation | Transformative learning and behavioral change |
| “I was surprised how much it changed me… I’m more conscious when I speak and act.” (P7) | Increased self-regulation | Internalised learning | Transformative learning and behavioral change |
| “It’s not just work. Now I’m more patient with my family now too.” (P8) | Transfer to personal life | Spillover effect | Transformative learning and behavioral change |
| “The horse doesn’t follow commands, it follows how you lead.” (P2) | Leading through presence | Leadership alignment and intentionality | Transformative learning and behavioral change |
| “Once I gained the horse’s trust, I realized I need to earn the same trust from my followers at work.” (P5) | Trust-building | Building rapport and relational trust | Relational leadership and trust-based engagement |
| “Now my team opens up to me more as I think it’s because I lead with more patience.” (P3) | Empathy and patience | Relational engagement | Relational leadership and trust-based engagement |
| “I’ve noticed that the team dynamic is more cooperative since I changed how I lead.” (P2) | Collaboration | Team collaboration | Relational leadership and trust-based engagement |
| “I try to show the same calm presence with my team that I learned from handling the horse.” (P7) | Modeling behavior | Leading by example | Relational leadership and trust-based engagement |
| “I no longer feel like I have to control everything. It’s more about mutual trust.” (P6) | Reduced control / shared trust | Trust as a relational foundation | Relational leadership and trust-based engagement |
| “I think my team can sense when I’m more grounded. That changes how they respond to me.” (P8) | Leader emotional presence | Relational influence | Relational leadership and trust-based engagement |
